# Thermodynamic basis for CFTR activity potentiation

**DOI:** 10.21203/rs.3.rs-7339733/v1

**Published:** 2025-08-12

**Authors:** Guangyu Wang

**Keywords:** allosteric coupling, digital biology, interdomain interaction, ligand modulation, thermodynamic signature, least-stable interaction, melting threshold, thermoring, protein stability

## Abstract

Trikafta modulators can correct the thermal and gating defects of the most common cystic fibrosis mutant F508del of the human cystic fibrosis transmembrane conductance regulator (hCFTR). While folding correctors VX-445 and VX-809 are sufficient to restore the Mg/ATP-dependent dimerization between the two nucleotide binding domains (NBD1 and NBD2) for channel opening, the thermodynamic basis for the activity potentiation by VX-770 in Trikafta remains unknown. Here, the thermoring structures and interdomain interactions of NBD2 were examined and compared with the counterparts of NBD1 with or without F508 in response to ligand binding. The results demonstrated that comparable thermostability between dimerized NBD1 and NBD2 was required to stabilize an activated intermediate for the channel activity potentiation by VX-770. Thus, a global induced fit across the interdomain interfaces upon ligand binding may optimize cooperative ligand-mediated NBD dimerization and improve the treatment of cystic fibrosis.

## Introduction

Induced fit is a term used to describe the process in which an enzyme’s shape changes to fit its substrate during a reaction. An example of this can be seen in a metallo-dependent or class II Fructose-1,6-bisphosphate (FBP) aldolase from the extreme thermophile, *Thermus aquaticus* (Taq). This enzyme is a tetramer composed of two dimers. The loop region of each subunit can be in an open or closed conformation near the active site, facilitating the relocation of metal for keto bond polarization during catalysis (1). Another example is the multiple inositol polyphosphate phosphatases (MINPP) from the Gram-positive bacterium *Bifidobacterium longum* (BlMINPP). This enzyme has an α-domain polypeptide insertion called the U-loop, which is responsible for large ligand-driven conformational changes during the catalytic cycle, thermal stability, recovery of activity after heating, and kinetic parameters for hydrolysis of phytate (2).

Furthermore, a ligand-induced conformational change in a dimerization loop is crucial for epidermal growth factor (EGF) receptor dimerization and activation. An abnormal orientation between two loop-related domains can cause autoinhibition (3)

Induced fit has also been proposed to explain the gating mechanisms of the cystic fibrosis (CF) transmembrane conductance regulator (CFTR). The allosteric nature of this ligand-gated channel is combined with the enzymatic activity of an ATP-binding Cassette (ABC) transporter (4–7). Although the Mg/ATP-mediated dimerization between two nucleotide binding domains (NBD1 and NBD2) upon regulatory (R) domain phosphorylation by protein kinase A (PKA) is critical for coupling two transmembrane domains (TMD1 and TMD2) via intracellular loops (ICLs) for CFTR activation (8–12), the specific thermodynamic basis for the induced fit across the interdomain interfaces is still missing.

Both NBD1 and NBD2 in hCFTR have highly conserved sequences and motifs at a dimerization interface (13–14). In an ATP-binding “head” subdomain, the aromatic ring of ATP is packed against W401 in NBD1 or Y1219 in NBD2 via a π−π interaction, and the remaining phosphate moiety is anchored by several parts via H-bonds. These parts include the Walker A motif (^458^GSTGAGKTS^466^ in NBD1 and ^1244^GRTGSGKST^1252^ in NBD2), the Walker B motif (from R560 to D572 and S573 in NBD1 or from R1358 to D1370 and E1371 in NBD2), and the switch regions (Q-loop involving Q493 in NBD1 or Q1291 in NBD2 while H-loop containing S605 in NBD1 or H1402 in NBD2). In the α-helical (or “tail”) subdomain, the conserved ATP-binding cassette (ABC) signature (^548^LSGGQ^552^ in NBD1 but ^1346^LSHGH^1350^ in NBD2), together with the Walker, is also needed to sandwich ATP. Based on this head-to-tail dimerization configuration, the two ATP-binding sites are inherently asymmetric because only E1371 and H1402 at site 2 rather than S573 and S605 at site 1 can hydrolyze ATP (15–19).

The cryo-electron microscopy (cryo-EM) structure of human CFTR revealed that NBD1 and NBD2 are separated by the R domain in the dephosphorylated closed state (PDB, 5UAK). NBD1 contains a disordered regulatory insertion (RI) (spanning residues 405–436) and an unstructured regulatory extension (RE) (spanning residues 647–678), which are unique to CFTR compared to other ABC transporters. On the other hand, NBD2 maintains its entire structure within a peptide range from 1207 to 1436 (8). Upon R domain phosphorylation and Mg/ATP-binding (PDB, 6MSM), NBD1 and NBD2 dimerize. In this state, the unstructured RI in NBD1 shortens to a segment from 410 to 434, while the disordered RE in NBD1 begins at residue 638. Meanwhile, structured NBD2 expands from 1202 to 1451 (9).

On the other hand, when the most common cystic fibrosis-causing F508 deletion destabilizes NBD1 (20–25), the ATP-dependent NBD dimerization is compromised (26). Although a combination of folding correctors elexacaftor/VX-445 and lumacaftor/VX-809 is enough to restore the NBD dimerization (27), the activity potentiator ivacaftor/VX-770 in Trikafta is still necessary to maximize the F508del activity (28, 29). Given that the additional binding of VX-770 to F508del-CFTR with VX-445, VX-661 bound fail to further enhance the ICL4-NBD1-NBD2 and TMD1-TMD2 interactions (30, 31), how VX-770 potentiates the channel activity is still unknown.

In this study, a highly-sensitive grid thermodynamic model, which was recently developed and examined (30–39), was used to test a hypothesis that comparable thermostability between Mg/ATP-dimerized NBD1 and NBD2 is required for maximal channel activity. To confirm this hypothesis, the VX-770-induced changes in the thermoring structures of NBD2 and the ICL2-NBD2 interactions of hCFTR/E1371Q and (F508del)hCFTR/E1371Q were further analyzed and compared with the counterparts of NBD1 in tightly dimerized or partially dimerized states. The results showed that the binding of VX-770 to hCFTR with or without F508 significantly induced comparable thermostability between Mg/ATP-dimerized NBD1 and NBD2, stabilizing an activated intermediate for maximal channel activity with a minimal activation energy barrier. Therefore, a global induced fit across the interdomain interfaces was still necessary to rescue the gating defect of the F508del mutant.

## Results

### Melting threshold of the dimerized NBD2 in phosphorylated (E1371Q)hCFTR with Mg/ATP bound matched the thermal inactivation temperaqture of 45°C

When (E1371Q)hCFTR is phosphorylated for Mg/ATP-dependent NBD1-NBD2 dimerization, the NBD2 (PDB, 6MSM) is structured from D1202 to P1451 but becomes disordered after P1451 (9). In this case, it is worth examining if NBD2 has a melting threshold (T_m,th_) that is comparable to that of NBD1.

**Figure 1:**
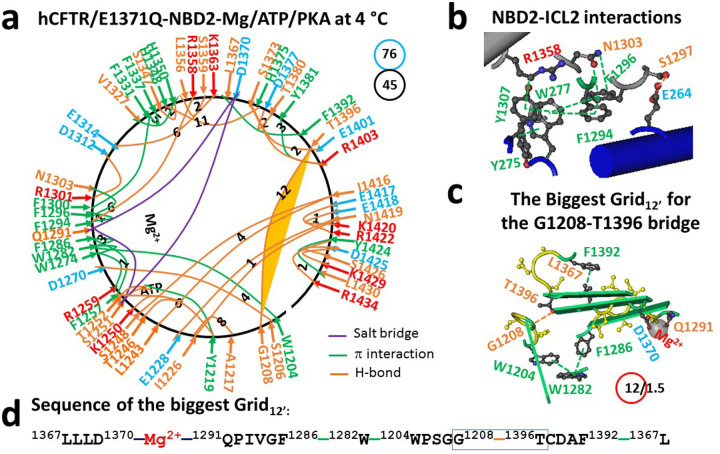
The thermoring structures of NBD2 in phosphorylated hCFTR/E1371Q with F508 in an activated intermediate at 4 °C. (**a**) The grid-like noncovalently interacting mesh network of NBD2 based on the cryo-EM structure of hCFTR/E1371Q with F508 in the presence of Mg/ATP/PKA at 4 °C (PDB ID, 6MSM, 3.2 Å). No software was used to create the image. Salt bridges, H-bonds and pi interactions are colored purple, orange, and green, respectively. The constrained grid sizes required to control the least-stable noncovalent interactions in the grids are labeled with black numbers. The least-stable G1208-T1396 H-bond in the biggest Grid_12_ is highlighted. The total grid sizes and the total grid size-controlled noncovalent interactions along the single peptide chain of NBD2 from D1202 to P1451 are shown in cyan and black circles, respectively. (**b**) The NBD2-ICL2 interactions. (**c**) The structure of the biggest Grid_12_ with a 12-residue size to control the least-stable G1208-T1396 H-bond. The grid size and the equivalent basic H-bonds for the least-stable noncovalent interaction are shown in and near a red circle. (**d**) The sequence of the biggest Grid_12_ to control the least-stable G1208-T1396 H-bond in the blue box.

Similar to the normal Mg/ATP binding site in NBD1 (30), a stable and rigid triangle was formed by S1251, Q1291 and D1370 via Mg^2+^ in NBD2. Meanwhile, ATP connected Y1219, T1246, K1250 and T1252 (Fig. 1a–b, Table S1). Notably, in addition to the H1348-H1375 H-bond between α and β subdomains, I1226-N1419-E1228 and T1246-I1416-S1248 H-bonds appeared between N- and C-termini (Fig. 1a). When the total numbers of noncovalent interactions and grid sizes were 45 and 76 (Table S1), respectively, the systematic thermal instability (T_i_) of NBD2 was 1.69 in 6MSM (Table 1), lower than the NBD1’s 1.88 in the same construct (30).

At the NBD2-ICL2 interface, in addition to the E264-S1297 H-bond, seven π interactions such as R1358-Y1307-W277-F1296 and N1303-F1296-F1294-W277-Y275 emerged. Similar to the triangle formed by F508, Y1073 and F1074 at the NBD1-ICL4 interface (30), F1296, W277, and F1294 also formed the smallest triangle to stabilize or rigidize the NBD2-ICL2 interface in this activated intermediate (Fig. 1b).

Consequently, although the weakest Q525-E585 H-bond between the α and β subdomains finalizes the posttranslational folding of NBD1 (31), the least-stable H-bond between the side chain of T1396 in the β-subdomain and the backbone C = O of G1208 in the N-terminal subdomain was responsible for the final posttranslational folding of NBD2 (Fig. 1a). It was controlled by the biggest Grid_12_ via a thermoring from L1367, D1370, Mg^2+^, Q1291, F1286, W1282, W1204, G1208, T1396, F1392, and back to L1367 (Figs. 1c–d). For 1.5 equivalent basic H-bonds to seal it, the calculated T_m,th_ of NBD2 in the 6MSM structure was approximately 45°C (Table 1), lower than the T_m,th_ of 50°C for NBD1 in the same structure (30). Therefore, the thermostability between the dimerized NBD1 and NBD2 in hCFTR was not comparable upon Mg/ATP binding. Given that the predicted T_m,th_ of 45°C of NBD2 was in excellent agreement with the experimental inactivation temperature of WT hCFTR (Table 1) (40), the thermal inactivation may result from the heat unfolding of the weakest G1208-T1396 H-bond of NBD2.

### Comparable thermostability between the dimerized NBD1 and NBD2 in phosphorylated (E1371Q)hCFTR with Mg/ATP/VX770 bound

**Figure 2:**
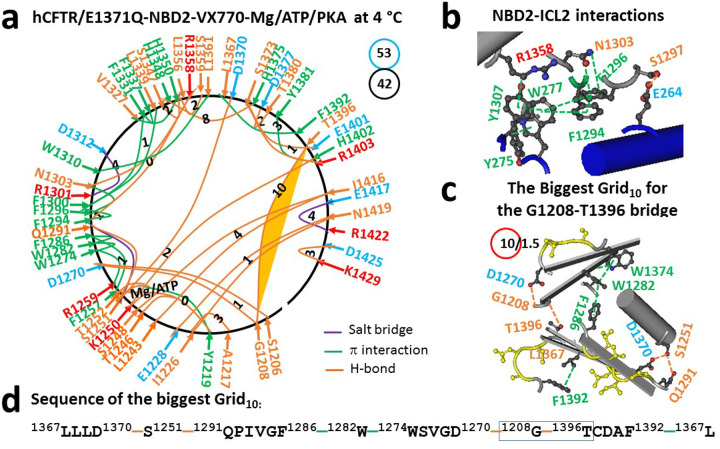
The thermoring structures of NBD2 in phosphorylated hCFTR/E1371Q/VX770 with F508 in an activated intermediate at 4 °C. (**a**) The grid-like noncovalently interacting mesh network of NBD2 based on the cryo-EM structure of hCFTR/E1371Q with F508 in the presence of Mg/ATP/PKA/VX-770 at 4 °C (PDB ID, 6O2P, 3.3 Å). No software was used to create the image. Salt bridges, H-bonds and pi interactions are colored purple, orange, and green, respectively. The constrained grid sizes required to control the least-stable noncovalent interactions in the grids are labeled with black numbers. The least-stable G1208-T1396 H-bond in the biggest Grid_10_ is highlighted. The total grid sizes and the total grid size-controlled noncovalent interactions along the single peptide chain of NBD2 from D1202 to P1451 are shown in cyan and black circles, respectively. (**b**) The NBD2-ICL2 interactions. (**c**) The structure of the biggest Grid_10_ with a 10-residue size to control the least-stable G1208-T1396 H-bond. The grid size and the equivalent basic H-bonds for the least-stable noncovalent interaction are shown in and near a red circle. (**d**) The sequence of the biggest Grid_10_ to control the least-stable G1208-T1396 H-bond in the blue box.

Along with additional VX-770 binding to the TMD1/TMD2 interface to enhance CFTR activity (41–45), the same NBD2-ICL2 interactions were also associated with the weakest G1208-T1396 H-bond (Fig. 2a–b, Table S2). However, this H-bond was controlled by the biggest Grid_10_ via a thermoring from L1367 to D1370, S1251, Q1291, F1286, W1282, W1274, D1270, G1208, T1396, F1392 and back to L1367 (Fig. 2c–d). When this H-bond was energetically equivalent to 1.5 basic H-bonds (1.5 kcal/mol), the calculated T_m,th_ of NBD2 in 6O2P was about 49°C (Table 1), similar to the T_m,th_ of 50°C for NBD1 in 6O2P (31). Therefore, the comparable thermostability between NBD1 and NBD2 was observed with their dimerization induced by Mg/ATP/VX770 binding in the activated intermediate to maximize channel activity. Meanwhile, along with a decrease in the totals of noncovalent interactions and grid sizes from 45 and 76 in 6MSM to 42 and 53 in 6O2P, the systematic thermal instability (Ti) of NBD2 also declined from 1.69 to 1.26 (Table 1).

Given that Trikafta contains the potentiator VX-770 and significantly enhances the activity of (F508del)hCFTR (28–29), it is intriguing to explore whether tight NBD dimerization induced by Mg/ATP/Trikafta also corresponds to balanced thermostability for both NBD1 and NBD2 even in the presence of the destabilizing F508 deletion.

### Matched thermostability between the dimerized NBD1 and NBD2 in phosphorylated (E1371Q/F508del)hCFTR with Mg/ATP/Trikafta bound

**Figure 3:**
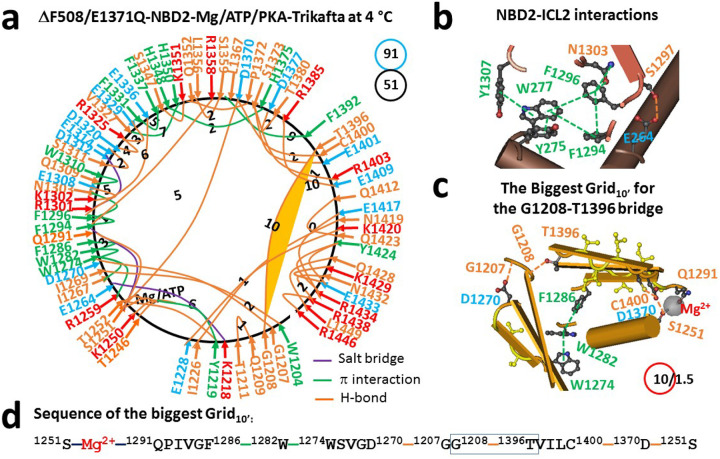
The thermoring structures of NBD2 in phosphorylated hCFTR/E1371Q/DF508 with Trikafta bound in an activated intermediate at 4 °C. (**a**) The grid-like noncovalently interacting mesh network of NBD2 based on the cryo-EM structure of hCFTR/E1371Q/DF508 with Trikafta bound in the presence of Mg/ATP/PKA at 4 °C (PDB ID, 8EIQ, 3.2 Å). No software was used to create the image. Salt bridges, H-bonds and pi interactions are colored purple, orange, and green, respectively. The constrained grid sizes needed to control the least-stable noncovalent interactions in the grids are labeled with black numbers. The least-stable G1208-T1396 H-bond in the biggest Grid_10’_ is highlighted. The total grid sizes and the total grid size-controlled noncovalent interactions along the single peptide chain of NBD2 from I1203 to P1451 are shown in cyan and black circles, respectively. (**b**) The NBD2-ICL2 interactions. (**c**) The structure of the biggest Grid_10’_ with a 10-residue size to control the least-stable G1208-T1396 H-bond. The grid size and the equivalent basic H-bonds for the least-stable noncovalent interaction are shown in and near a red circle. (**d**) The sequence of the biggest Grid_10’_ to control the least-stable G1208-T1396 H-bond in the blue box.

When F508 was deleted, both NBD1 and NBD2 changed their thermoring structures to maintain Mg/ATP-dependent dimerization in the presence of Trikafta (Figs. 3a; Table S3) (30). In addition to the intact ATP site and the compromised S1251-Mg^2+^-Q1291 bridge along with the broken R1358-Y1307 CH-π interaction at the NBD2-ICL2 interface (Fig. 3a–b), only a small fraction of 37 noncovalent interactions were conserved in NBD2. For example, the R1259-W1274-W1282-F1286, F1294-F1296-N1303, V1327-H1350, F1331-F1337 and L1367-F1392 π interactions, together with the Y1219-T1252 and S1373-E1401 H-bonds. Despite these changes, the G1208-T1396 H-bond between N-terminal and β- subdomains was still the weakest to finalize the posttranslational NBD2 folding (Fig. 3a). However, it was governed by another biggest Grid_10’_ via a thermoring from S1251, Mg^2+^, Q1291, F1286, W1282, W1274, D1270, G1207, G1208, T1396, C1400, D1370, and back to S1251 (Fig. 3c–d). Since the weakest G1208-T1396 H-bond was still energetically equivalent to 1.5 basic H-bonds, the calculated melting threshold (T_m,th_) was 49°C (Table 1), which was exactly the same as the 49°C of NBD1 in the same construct (30). On the other hand, when the total numbers of noncovalent interactions and grid sizes increased from 42 and 53 to 51 and 91, respectively, the systematic thermal instability (T_i_) of NBD2 also increased from 1.26 to 1.78 (Table 1).

**Figure 4:**
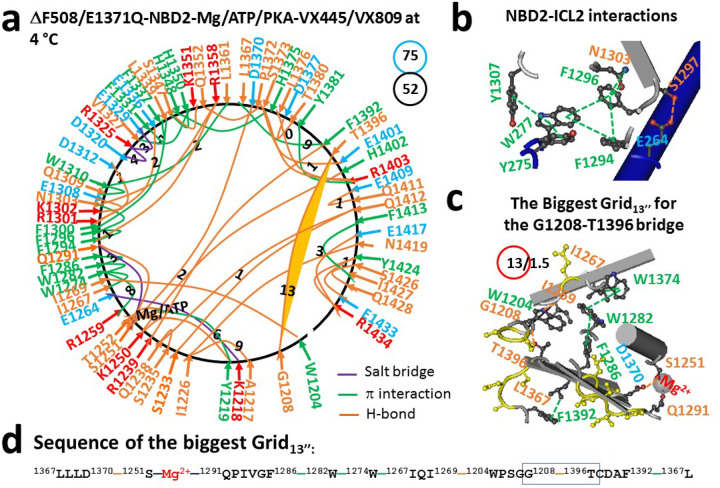
The thermoring structures of NBD2 in phosphorylated hCFTR/E1371Q/DF508 with VX-445/VX809 bound in an activated intermediate at 4 °C. (**a**) The grid-like noncovalently interacting mesh network of NBD2 based on the cryo-EM structure of hCFTR/E1371Q/DF508 with VX-445/VX809 bound in the presence of Mg/ATP/PKA at 4 °C (PDB ID, 8EIO, 2.8 Å). No software was used to create the image. Salt bridges, H-bonds and pi interactions are colored purple, orange, and green, respectively. The constrained grid sizes needed to control the least-stable noncovalent interactions in the grids are labeled with black numbers. The least-stable G1208-T1396 H-bond in the biggest Grid_13_ is highlighted. The total grid sizes and the total grid size-controlled noncovalent interactions along the single peptide chain of NBD2 from I1203 to P1451 are shown in cyan and black circles, respectively. (**b**) The NBD2-ICL2 interactions. (**c**) The structure of the biggest Grid_13_ with a 13-residue size to control the least-stable G1208-T1396 H-bond. The grid size and the equivalent basic H-bonds for the least-stable noncovalent interaction are shown in and near a red circle. (**d**) The sequence of the biggest Grid_13_ to control the least-stable G1208-T1396 H-bond in the blue box.

In contrast, when Trikafta was replaced with VX-445 and VX-809 in 8EIO, the weakest G1208-T1396 H-bond in dimerized NBD2 was governed by the biggest Grid_13_ via a thermoring from L1367 to D1370, S1251, Mg^2+^, Q1291, F1286, W1282, W1274, I1267, I1269, W1204, G1208, T1396, F1392, and back to L1367 (Fig. 4; Table S4). Since this H-bond was energetically equivalent to 1.5 basic H-bonds (1.5 kcal/mol), the calculated T_m,th_ of NBD2 in 8EIO was 43°C (Table 1), which was 4°C lower than the T_m,th_ of 47°C for NBD1 in the same construct (31). Notably, despite a decrease in the T_m,th_ of NBD2, the systematic thermal instability (Ti) of NBD2 reduced from 1.78 in 8EIQ to 1.44 in 8EIO (Table 1).

#### Significant imbalance in thermostability between partially dimerized NBD1 and NBD2 in phosphorylated (F508del)hCFTR/E1371Q with only Mg/ATP/elexacaftor bound

To further investigate the comparable thermostability between NBD1 and NBD2 as a requirement for Trikafta to maximize the channel activity of the F508del mutation, it is necessary to determine if their comparable thermostability would be significantly compromised in a partially dimerized state.

**Figure 5:**
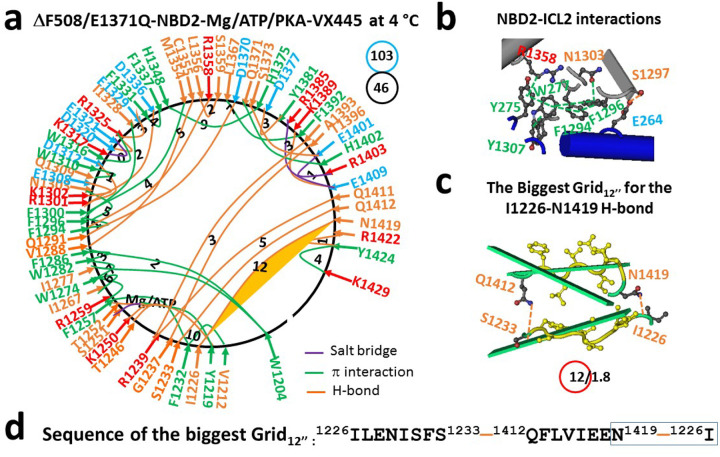
The thermoring structures of NBD2 in phosphorylated hCFTR/E1371Q/DF508 with Mg^2+^/ATP/elexacaftor (VX445) bound in the closed state at 4 °C. (**a**) The grid-like noncovalently interacting mesh network of NBD2 based on the cryo-EM structure of hCFTR/E1371Q/DF508 with elexacaftor bound in the presence of Mg/ATP/PKA at 4 °C (PDB ID, 8EIG, 3.7 Å). No software was used to create the image. Salt bridges, H-bonds and pi interactions are colored purple, orange, and green, respectively. The constrained grid sizes necessary to control the least-stable noncovalent interactions in the grids are labeled with black numbers. The least-stable I1226-N1419 H-bond in the biggest Grid_12’_ is highlighted. The total grid sizes and the total grid size-controlled noncovalent interactions along the single peptide chain of NBD2 from I1203 to K1429 are shown in cyan and black circles, respectively. (**b**) The NBD2-ICL2 interactions. (**c**) The structure of the biggest Grid_12’_ with a 12-residue size to control the least-stable I1226-N1419 H-bond. The grid size and the equivalent basic H-bonds for the least-stable noncovalent interaction are shown in and near a red circle. (d) The sequence of the biggest Grid_12’_ to control the least-stable I1226-N1419 H-bond in the blue box.

When only elexacaftor and Mg^2+^/ATP are bound to phosphorylated closed (E1371Q/F508del)hCFTR, NBD1 and NBD2 are partially dimerized (27). In this scenario, the total numbers of noncovalent interactions and grid sizes of NBD2 changed from 52 and 75 in 8EIO to 46 and 103 in 8EIG, respectively (Fig. 5a; Table S5). Hence, the systematic thermal instability (T_i_) of NBD2 significantly increased from 1.44 in 8EIO to 2.24 in 8EIG (Table 1).

Notably, the least-stable G1208-T1396 H-bond in the biggest Grid_13_ was replaced by the least-stable I1226-N1419 H-bond in the biggest Grid_12’_ of NBD2 while the R1358-Y275 π interaction was reestablished at the NBD2-ICL2 interface (Fig. 5a–b). This biggest thermoring cycled from I1226 to S1233, Q1412, N1419, and back to I1226 (Fig. 5c–d). Since this H-bind was energetically equivalent to 1.8 basic H-bonds, the calculated T_m,th_ of NBD2 in 8EIG was about 48°C, significantly 9°C higher than the T_m,th_ of 39°C in NBD1 (30). Therefore, comparable thermostability between Mg/ATP-dimerized NBD1 and NBD2 was indeed required for Trikafta to maximize the channel activity of the F508del mutant.

## Discussion

Interdomain interactions play a crucial role in regulating CFTR activity, yet little is known about how each domain responds to different physical, chemical and genetic stimuli during this process. This study revealed that while the apparent secondary structures of dimerized NBD1 and NBD2 remained the same or similar before vs. after the introduction of the F508 deletion along with various folding correctors, their tertiary thermoring structures underwent a global change, rearranging interdomain interactions. Furthermore, although the additional binding of VX770 to hCFTR or F508del with folding correctors VX445 and VX-809 did not enhance interdomain interactions, a global induced fit across domain-domain interfaces significantly triggered a conformational selection step, ensuring comparable thermostability between Mg/ATP-dimerized NBD1 and NBD2 to stabilize the activated intermediate with a minimal activation energy barrier for maximal channel activity of hCFTR, regardless of the presence of F508del.

### Comparable thermostability between the dimerized NBD1 and NBD2 upon VX-770 binding maximizes hCFTR activity

**Figure 6:**
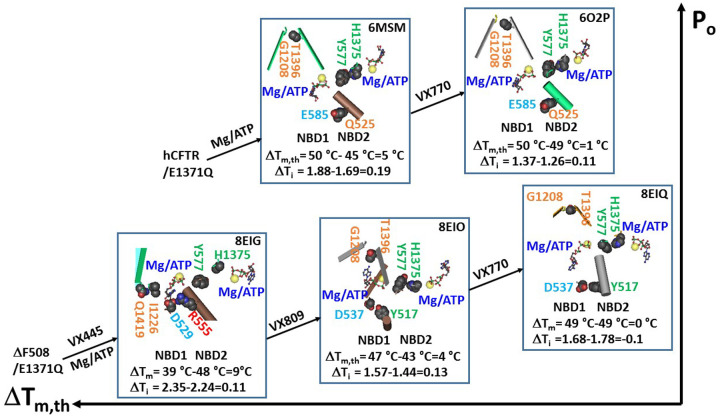
Thermostability comparison between NBD1 and NBD2 upon Mg/ATP-induced dimerization for CFTR activation. Cryo-EM structures of phosphorylated hCFTR/E1371Q with Mg/ATP bound (PDB: 6MSM), phosphorylated hCFTR/E1371Q with Mg/ATP/VX-770 bound (PDB: 6O2P), phosphorylated hCFTR/DF508/E1371Q with Mg/ATP/elexacaftor bound (PDB: 8EIG), phosphorylated hCFTR/DF508/E1371Q with Mg/ATP/VX-445/VX-809 bound (PDB: 8EIO), and phosphorylated hCFTR/DF508/E1371Q with Mg/ATP/Trikafta bound (PDB: 8EIQ) in a closed or activated intermediate are used for the model. The Y577-H1375 pi interaction at the NBD1-NBD2 interface acts as a dimerization hallmark. The calculated melting thresholds (T_m,th_) and systematic thermal instabilities (T_i_) of NBD1 and NBD2 and their differences are shown below them.

In this study, despite the tight NBD1-ICL4 or NBD2-ICL2 interactions still allowing for intact Mg/ATP binding sites in the phosphorylated activated intermediate of hCFTR/E1371Q (6MSM) (Figs. 1a–b) (30), both dimerized NBD1 and NBD2 exhibited different melting thresholds (T_m,th_) of 50°C and 45°C, respectively (Fig. 6). Given that the WT hCFTR channel starts thermal inactivation at 45°C (40), the inactivation may be due to the unfolding of least-stable G1208-T1396 bridge in NBD2 at the T_m,th_ of 45°C (Table 1, Fig. 6). When the potentiator VX-770 was bound to the E1371Q mutant increasing channel activity, the dimerized NBD2 increased the T_m,th_ from 45°C to 49°C (Fig. 6). Thus, although VX-770 still potentiates the WT, G551D and W1282X-CFTR activity in a phosphorylation-dependent but ATP-independent manner (44–46), VX-770-induced comparable thermostability between dimerized NBD1 and NBD2 favors the maximal activity of hCFTR in an ATP-dependent manner.

### Matching thermostability between dimerized NBD1 and NBD2 upon Trikafta binding maximizes (F508del)hCFTR activity

In the previous study, two identical isolated hNBD1-Δ(RI, RE) or (F508del)hNBD1-Δ(RI,RE) constructs with the same thermostability can form a stable homodimer (2PZE or 2PZF) for crystal capture upon Mg/ATP binding, suggesting that the RI or RE may serve as an important dimerization loop or segment (20, 21). In this study, despite the instablity of NBD1 in ΔF508 (27), tightly dimerized NBD1 and NBD2 upon Trikafta binding to TMD1 and TMD2 still shared a common T_m,th_ of 49°C (Fig. 6) (30). However, in the presence of elexacaftor and lumacaftor, the T_m.th_ of NBD1 was 47°C, slightly higher than the T_m.th_ of 43°C in NBD2 (Fig. 6). Further, the T_m,th_ values of the partially-dimerized NBD1 and NBD2 in phosphorylated (F508del)hCFTR/E1371Q with elexacaftor bound were 39°C and 48°C, respectively (Fig. 6) (30). Therefore, matching thermostability between dimerized NBD1 and NBD2 upon Trikafta binding facilitates correcting the thermal and gating defects of the F508del mutation.

### Asymmetric weakest noncovalent bridges across the dimerization interface

Although both tightly dimerized NBD1 and NBD2 exhibited minimal differences in T_m,th_ under two various conditions, they had distinct but asymmetric weakest noncovalent bridges (Fig. 6). When the potentiator VX-770 was bound to hCFTR/E1371Q, the biggest Grid_8_ in dimerized NBD1 was found to be responsible for the least-stable Q525-E585 H-bond between α and β-subdomains (31). However, the biggest Grid_10_ in dimerized NBD2 was responsible for the least-stable G1208-T1396 H-bond between C- and N-termini (Figs. 2a, 6). After Trikafta modulators are bound to the E1371Q/F508del mutant, the least-stable Y517-D537 H-bond finalized posttranslational NBD1 folding (30). In contrast, the weakest G1208-T1396 H-bond was still the last step of the posttranslational NBD2 folding (Figs. 3a, 6). Since this weakest link was also the final posttranslational CFTR folding, its stability is essential for optimizing CFTR activity and cystic fibrosis treatment.

Taken together, while folding modulators in Trikafta shift the weakest tertiary link to serve as the final posttranslational NBD1 folding event, they do not impact the final posttranslational NBD2 folding. Conversely, the potentiator VX-770 in Trikafta is essential for the stabilized activated intermediate to maximal channel activity by adjusting the size of the biggest thermoring in NBD2 to align with the thermal stability of NBD1. Therefore, these three modulators in Trikafta play distinct roles in addressing thermal and gating abnormalities in (F508del)hCFTR (30, 31). In fact, in a cellular environment where Mg^2+^, ATP and PKA are present along with an allosteric drug like Trikafta or ivacaftor, the induced comparable thermostability between the dimerized NBD1 and NBD2 is essential for CFTR folding, stability and effective therapy, particularly when compromised by disease mutations such as F508del and G551D (20–27, 47–50).

## Conclusions

Induced fit plays a significant role in allosteric enzymatic reactions, especially in the case of CFTR. CFTR functions not only as an anion channel but also as an ATPase. In the CFTR gating cycle, induced fit at the dimerization interface between NBD1 and NBD2 is crucial for an “interdependent protein dance”. When two Mg/ATP agonists bind normally at two interfacial sites, they dimerize NBD1 and NBD2 after R domain phosphorylation. A VX-770-inducd conformational selection step leads to an expanded induced fit between dimerized NBD1 and NBD2. As a result, their thermostability closes to each other to stabilize the activated intermediate with minimal activation energy barrier for maximal channel activity of hCFTR with or without F508del and folding correctors. Therefore, the increasing precision of thermostability evaluation permits increasing activation mechanistic detail and allosteric drug design to optimize the cystic fibrosis treatment.

## Computational Methods

### Data mining resources

Thermoring structures of phosphorylated and Mg/ATP bound hCFTR constructs with or without various modulators in the activated intermediate were analyzed using cryo-EM structures at 4°C. The structures without F508del included hCFTR/1371Q with Mg/ATP bound (PDB ID, 6MSM, model resolution = 3.2 Å) (9), and with Mg/ATP/VX-770 bound (PDB ID, 6O2P, model resolution = 3.3 Å) (40). Furthermore, the structures with F508del covered hCFTR/E1371Q/ΔF508 with Mg/ATP/ elexacaftor (VX445) bound (PDB ID, 8EIG, model resolution = 3.7 Å), Mg/ATP/VX445/VX809 bound (PDB ID, 8EIO, model resolution = 2.8 Å), and Mg/ATP/Trikafta bound (PDB ID, 8EIQ, model resolution = 3.2 Å) (27).

### Standard methods for filtering tertiary non-covalent interactions

Tertiary non-covalent interactions such as salt bridges, H-bonds and π interactions in NBD2 were filtered using standard methods and precise calculations previously employed to ensure accurate and reproducible results (30–39). Detailed cutoff distances and interaction angles (for an H-bond) can be found in the online Supplementary Information (Tables S1, S2, S3, S4 and S5). It shoud be noted that momentary fluctuation-induced perturbations in noncovalent interactions during protein dynamics were not considered in this study. Thus, approximately 42–52 noncovalent interactions were identified along the single peptide chain from D1202 to P1451 in NBD2 of each protomer.

### Calculations based on the thermoring structures and the grid thermodynamic model

The previously established grid thermodynamic model was used to map the systematic fluidic grid-like noncovalent interaction mesh networks of NBD1 and NBD2 (30–39). In these networks, identified noncovalent interactions and linked amino acid residues were represented by edges and nodes, respectively. When a noncovalent interaction had a direct zero-length path between two linked nodes and the shortest reverse path from one node back to the other through other noncovalent interactions and a peptide segment, a thermoring or grid with the tightest network was formed to control this least-stable noncovalent interaction within it. The grid size was defined as the free or silent residues not involved in any noncovalent interactions along the shortest reverse path.

In this way, each thermoring acted like a bow and each least-stable noncovalent interaction functioned like a vibrating bowstring. Thus, the strength of each bowstring was determined not only by itself but also regulated by the bow length, the relevant energy relocation and allosteric propagation. Generally, the intensity of a noncovalent interaction or a bowstring was limited to 1–3 kcal/mol. However, the more free or flexible side chains along the thermoring or the bow, or the larger the thermoring size or the longer the bow, the weaker the controlled noncovalent interaction or the bowstring.

Once the biggest grid was identified, the least-stable noncovalent interaction within it was typically the weakest one along the entire polypeptide chain. Its heat unfolding could be characterized by a specific melting temperature threshold using the following equation as previously examined (30–39):

(1) $${\text{T}}_{\text{m},\text{th}}({}^{\circ}\text{C})=34+(\text{n}-2)\times 10+(20-\text{s})\times 2$$

where, n represents the total number of basic H-bonds (each approximately 1 kcal/mol) equivalent to the least-stable noncovalent interaction controlled by the grid; and s is the grid size used to control the least-stable noncovalent interaction within the grid. In this study, although the I1226-N1419 H-bond was highly conserved in NBD2, it was not the weakest or least-thermostable except in 8EIG. For example, because E1228 also H-bonded to N1419 via their side chains in 6MSM, it was actually controlled by the smaller Grid_1_ via a smaller thermoring from I1226 to E1228, N1419, and back to I1226 and the T_m,th_ to unfold it was at least 72°C (Figs. 1a).

In addition to the T_m,th_, the total grid sizes (S) and the total non-covalent interactions (N) along the same polypeptide chain could be utilized to calculate grid-based systematic thermal instability (T_i_) using the same equation as examined previously (30–39):

(2) $${\text{T}}_{\text{i}}=\text{S}/\text{N}$$

This parameter reflects the peptide’s compact conformational entropy or flexibility.

## Supplementary Material

Supplementary Files

This is a list of supplementary files associated with this preprint. Click to download.

- SupplementaryInformationCFTRpotentiationmechanismv1.6.pdf

**Supplementary Information** The online version contains supplementary material available.

## Figures and Tables

**Table 1:** Grid thermodynamic model-based new parameters of NBD2.

Construct	hCFTR/E1371Q				
PDB ID	6MSM	6O2P	8EIG	8EIO	8EIQ
F508	+	+	−	−	−
Mg/ATP	+	+	+	+	+
Phosphorylation	+	+	+	+	+
Sampling temperature, °C	4	4	4	4	4
Tight NBD dimerization	+	+	−	+	+
NBDi	2	2	2	2	2
Normal Mg^2+^ site	+	−	−	−	−
Name of the biggest grid	Grid_12_	Grid_10_	Grid_12’_	Grid_13_	Grid_10’_
Grid size (s)	12	10	12	13	10
# of energetically equivalent basic H-bonds (n) controlled by Grid_s_	1.5	1.5	1.8	1.5	1.5
Total non-covalent interactions (N)	45	42	46	52	51
Total grid sizes (S), a.a.	76	53	103	75	91
Systematic thermal instability (T_i_)	1.69	1.26	2.24	1.44	1.78
Calculated T_m,th_, °C	**45**	49	48	43	49
Measured inactivation temperature, °C	**45**				
Ref. for measured T_inact_	(40)				

## Data Availability

Data are provided within the manuscript or supplementary information files

## Abbreviations

ABC: ATP-binding cassette
BlMINPP: Gram-positive bacterium *Bifidobacterium longum*
CF: cystic fibrosis
cryo-EM: cryoelectron microscopy
CFTR: cystic fibrosis transmembrane conductance regulator
DSC: differential scanning calorimetry
EGF: epidermal growth factor
FBP: Fructose-1,6-bisphosphate
hCFTR: human CFTR
ICL2: intracellular loop 2
ICL3: intracellular loop 3
ICL4: intracellular loop 4
MINPP: multiple inositol polyphosphate phosphatases
NBD1: nucleotide binding domain 1
NBD2: nucleotide binding domain 2
PKA: protein kinase A
R: regulatory
RE: regulatory extension
RI: regulatory insert
T_i_: systematic thermal instability
T_m,th_: melting temperature threshold
Taq: Thermus aquaticus
TMD1: transmembrane domain 1
TMD2: transmembrane domain 2
WT: wild type
